# Anticancer effects of disulfiram: a systematic review of in vitro, animal, and human studies

**DOI:** 10.1186/s13643-021-01858-4

**Published:** 2022-06-02

**Authors:** Ling Wang, Yang Yu, Cong Zhou, Run Wan, Yumin Li

**Affiliations:** 1 Department of Gastric Cancer Surgery, Fudan University Shanghai Cancer Center, Shanghai, 200032 P.R. China; 2 Department of Oncology, Shanghai Medical College, Fudan University, Shanghai, 200032 P.R. China; 3 Key Laboratory of Digestive System Tumors of Gansu Province, Lanzhou University Second Hospital, Lanzhou, Gansu 730030 P.R. China; 4 Department of Tumor Surgery, Lanzhou University Second Hospital, Lanzhou, Gansu 730030 P.R. China; 5 Shaoxing People’s Hospital, Shaoxing, Zhejiang 312000 P.R. China

**Keywords:** Disulfiram, Apoptosis rate, Tumor inhibition rate, Progression-free survival, Overall survival

## Abstract

**Background and objectives:**

Cancer morbidity and mortality rates remain high, and thus, at present, considerable efforts are focused on finding drugs with higher sensitivity against tumor cells and fewer side effects. Disulfiram (DSF), as an anti-alcoholic drug, kills the cancer cells by inducing apoptosis. Several preclinical and clinical studies have examined the potential of repurposing DSF as an anticancer treatment. This systematic review aimed to assess evidence regarding the antineoplastic activity of DSF in in vitro and in vivo models, as well as in humans.

**Methods:**

Two authors independently conducted this systematic review of English and Chinese articles from the PubMed, Embase, and the Cochrane Library databases up to July 2019. Eligible in vitro studies needed to include assessments of the apoptosis rate by flow cytometry using annexin V/propidium iodide, and studies in animal models and clinical trials needed to examine tumor inhibition rates, and progression-free survival (PFS) and overall survival (OS), respectively. Data were analyzed using descriptive statistics.

**Results:**

Overall, 35 studies, i.e., 21 performed in vitro, 11 based on animal models, and three clinical trials, were finally included. In vitro and animal studies indicated that DSF was associated with enhanced apoptosis and tumor inhibition rates, separately. Human studies showed that DSF prolongs PFS and OS. The greatest anti-tumor activity was observed when DSF was used as combination therapy or as a nanoparticle-encapsulated molecule. There was no noticeable body weight loss after DSF treatment, which indicated that there was no major toxicity of DSF.

**Conclusions:**

This systematic review provides evidence regarding the anti-tumor activity of DSF in vitro, in animals, and in humans and indicates the optimal forms of treatment to be evaluated in future research.

**Supplementary Information:**

The online version contains supplementary material available at 10.1186/s13643-021-01858-4.

## Introduction

Cancer is expected to be the leading cause of death and the foremost contributor to decreased life expectancy in every country worldwide during the twenty-first century and beyond [1]. Although comprehensive therapies prolong survival and improve the quality of life of cancer patients, approximately 96,000,000 cancer deaths occurred in 2018 worldwide [1]. The global community is well aware that new drug development, discovery, and synthesis are a time-consuming process, which involves intensive work and appraisal of the cost-effectiveness of the drug under development [2]. As a result, researchers are allocating considerable efforts for repurposing existing drugs such as disulfiram (DSF).

In the 1800s, DSF was used as an industrial catalyst in the production of rubber [3]. In 1948, DSF was approved by the Food and Drug Administration for treating alcoholism [4]. In 1988, DSF was associated with a decrease in the occurrence of occasional infections in symptomatic patients with human immunodeficiency virus infection [5], prompting the conduct of a wealth of clinical trials, some of which are still ongoing (www.clinicaltrials.gov). The antineoplastic activity of DSF was first recorded in 1977 by Dr. Lewison in a 35-year-old female breast cancer patient with systemic metastases who received DSF for her severe alcoholic syndrome and remained clinically free of cancer for 10 years without receiving any form of anticancer therapy [6]. This observation was noted in an era in which the anticancer effect of DSF was being researched. In recent years, a large number of preclinical studies and clinical trials (www.clinicaltrials.gov) of DSF have been conducted to explore the anticancer activities of this drug. Nonetheless, the antitumor effectiveness of DSF remains uncertain owing to existing heterogeneity across different studies with cell lines, animals, and humans. Currently, a systematic review of these studies to assess and clarify the anticancer potential of DSF is lacking.

It is worthy to explore whether there are substantial differences and are appropriate for clinical proposals. Therefore, this study aimed to perform a systematic review of published data on the antitumor activity of DSF. Specifically, this review aimed to assess the apoptosis and tumor inhibition rates of DSF based on data from studies in cell lines and animal models, respectively, and examine the benefit of DSF on progression-free survival (PFS) and overall survival (OS) based on results from clinical studies, regardless of the study design or type of cancer investigated. Meanwhile, it is important for evaluating the anti-tumor effect of disulfiram to include in the side effects. The side effects of disulfiram will be covered in this article.

## Materials and methods

The Preferred Reporting Items for Systematic Reviews and Meta-Analyses guidelines were followed to conduct this systematic review [7].

### Search strategy

PubMed, Embase, and Cochrane Library databases were searched for relevant studies from their inception to the end of July 2019. The search was performed with a combination of Medical Subject Headings and free words as follows: (neoplasia OR neoplasm OR tumor OR cancer OR malignancy OR malignant neoplasm), and [disulfiram OR bis (diethylthiocarbamoyl) disulfide OR tetraethylthioperoxydicarbonic diamide OR tetraethylthiuram disulfide OR tetraethylthiuram OR antabus OR antabuse teturam OR dicupral OR esperal OR alcophobin OR anticol]. The details of the search strategy are presented in the supplement.

### Study selection

Studies that implemented the below criteria were included: (1) solid cancer cell lines, animals, or patients treated with DSF; (2) in vitro studies focusing on parameters of the apoptosis index (early apoptosis or early apoptosis plus late apoptosis) using annexin V-fluorescein isothiocyanate/propidium iodide double-staining analysis by flow cytometry, in vivo studies evaluating the tumor inhibition rate in cell-line-derived xenograft animal models, or studies in humans, which included OS and PFS as endpoints, to assess the effect of DSF in cancer patients; and (3) studies published in the English and Chinese language. There were no restrictions on the type of cancer studied. To avoid duplication of data, only the most recent and most comprehensive articles were included. Studies with incomplete data or conference abstracts were excluded. Two investigators (Ling Wang, Run Wan) independently screened the databases for studies based on the eligibility criteria. Any discrepancies were resolved by consulting a third researcher (Cong Zhou).

### Data derivation

Two investigators (Ling Wang, Cong Zhou) independently extracted data from the inclusive studies. Inconsistencies between the two investigators were resolved by consulting a third reviewer (Run Wan). When required, we contacted the authors of the research for further information. A pre-designed structured outline was used to abstract data. The outline included the following fields: study type (in vitro, in vivo, clinical study, or case series); general information (first author, publication year, country, and study design); supplement used; anticancer treatment used; and outcomes (i.e., apoptosis rate, tumor inhibition rate, OS and PFS, as applicable). The results of each study included were summarized. Descriptive statistics were used for data analysis. Meta-analysis was not performed owing to substantial heterogeneity across studies.

## Results

### Study characteristics

**Fig. 1:**
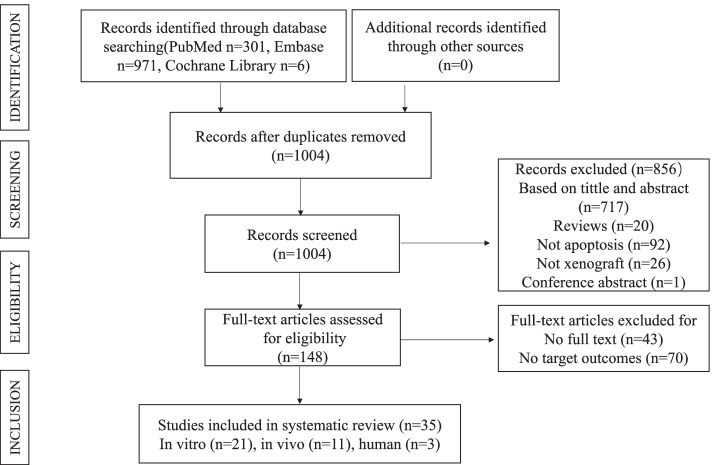
Flow diagram of literature search process

The initial search yielded a total of 1278 studies. After excluding 274 irrelevant and duplicate studies, the full texts of 1004 studies were screened. Of these, 148 were considered eligible based on the availability of full texts as well as the description of target outcomes. Ultimately, 113 articles were removed (no full texts, *n* = 43; no target outcomes, *n* = 70), and 35 studies were selected. A detailed description of the steps followed during the retrieval process is provided in Fig. 1.

Of 35 selected studies, 21 were in vitro studies (Table 1), 11 were in vivo studies with animal models (Table 2), and three were clinical trials (Table 3). In in vitro studies, the most studied cancer was breast cancer (five studies) [8–12], while the A549 non-small cell lung cancer (NSCLC) cell line was the one most commonly used cell line (four studies) [13–16]. Three studies examined DSF as a single agent [17–19], and 17 studies examined DSF in combination with metal ions (Cu, Ag), chemotherapy, or radiation therapy [8–16, 20–27]. In addition, DSF was encapsulated in nanoparticles (DSF-NPs) in three studies [12, 16, 25].

**Table 1:** Effects of disulfiram on cell apoptosis rates from in vitro studies

Reference	Country	Tumor	Percentage of apoptosis (%)						
			Intervention time	Negative control	Posivitive control	Cell lines	Negative control	Positive control	Treatment group
You et al.	China	Colorectal cancer	48 h	Saline	DOX (8.5 μM)	HCT116	0.27 ± 0.24	29.2 ± 4.1	DSF/Cu 0.05 μM: 8.55 ± 2.3, 0.1 μM: 24.02 ± 3.6, 0.2 μM: 38.4 ± 7.9, 0.4 μM: 58.3 ± 7.7
						HCT8	2.1±1.6	32.3 ± 4.1	DSF/Cu 0.05 μM: 29.5 ± 4.4, 0.1 μM: 28.1 ± 9.5, 0.2 μM: 38.6 ± 10.3, 0.4 μM: 56.4 ± 10.2
						SW620	2.21±0.5	48.4 ± 9.5	DSF/Cu 0.05 μM: 20.1±5.7, 0.1 μM: 30 ± 4.2, 0.2 μM: 42 ± 6.3, 0.4 μM: 43.45 ± 8.3
Yang et al.	Germany	Breast cancer	48 h	Control	CIS (5 μM)	MCF-7	25.31	31.67	DSF 1 μM: 36.6, DSF 1 μM + CIS 5 μM: 57.4
						MDA-MB-435S	5.843	5.447	DSF 1 μM: 13.56, DSF 1 μM + CIS 5 μM: 29.4
						SKB-R3	3.023	11.46	DSF 1 μM: 5.6, DSF 1 μM + CIS 5 μM: 7.71
Wu et al.	China	Triple-negative breast cancer	24 h	DMSO	PAX (5 nM)	SUM102 ALDH+	2.22	5.83	DSF/Cu 0.75 μM: 23.53
						SUM102 ALDH-	8.01	10.81	DSF/Cu 0.75 μM: 20.9
Guo et al.	Germany	Ovarian cancer	72 h	Control	_	IGROV1	10.32	_	Cu 1 μM: 15.3, DSF 1 μM: 25.46, DSF/Cu: 47.55
						SKOV3IP1	8.69		Cu 1 μM: 7.1, DSF 0.1 μM: 15.99, DSF/Cu: 55
						SKOV3	3.65		Cu 1 μM: 1.91, DSF 1 μM: 43.2, DSF/Cu: 50.4
Wu et al.	China	Non-small cell lung cancer	24 h	Control	_	A549	2.5	_	Cu 1 μM: 3.8, DSF 1.4 μM: 4.8, DSF/Cu: 35.4
						H460	4.7		Cu 1 μM: 3.7, DSF 8 μM: 4.9, DSF/Cu: 21.4
						H1299	8.7		Cu 1 μM: 10.3, DSF 4 μM: 7.1, DSF/Cu: 37.9
Chen et al.	China	Non-small cell lung cancer	24 h	Control	_	A549	3.35	_	Ag 1.25 μM: 4.34, DSF 1.25 μM: 5.14, DSF/Ag: 42.81
Butcher et al.	UK	Non-small cell lung cancer	16 h	Vehicle	_	A549	6.3	_	CuCl_2_ 10 μM: 6.5, DSF 1 μM: 15.2, DSF/CuCL_2_: 47.2
Albers et al.	Germany	Head and neck squamous	48 h	Control	CIS (1μM)+10Gy	HNSCC cell lines	11.35	CIS 1 μM: 24.12, 10Gy: 23.47	DSF 3 μM/Cu 0.1 μM: 20.87, DSF 3 μM + CIS 1 μM: 38.35, DSF 3 μM/Cu 0.1 μM + CIS 1 μM: 51
		cell carcinoma						CIS 1 μM + 10Gy: 30.68	DSF 3 μM: 17.66, CIS 1 μM + 10Gy+ DSF 3 μM: 44.82, CIS 1 μM + 10Gy+ DSF 3 μM/Cu 0.1 μM: 61.5
Yang et al.	China	Nasopharyngeal cancer	6 h	Control	_	CNE-2Z	4.41	_	DSF 0.2 μM/Cu 10 μM: 24.08, DSF 0.4 μM/Cu 10 μM: 58.2
						NP69-SV40T	0.55	_	DSF 0.2 μM/Cu 10 μM: 1.19, DSF 0.4 μM/Cu 10 μM: 5.99
Marwa et al.	Egypt	Colon cancer	72 h	Control	_	DCECs	1.58	_	DSF 9.5 ± 0.9 μg/mL: 60.31 ± 1.2, UC-NPs 1548.7 ± 25 μg/mL: 12.12 ± 0.47, C-NPs 3122.4 ± 39 μg/mL: 2.6 ± 0.07
						CDCECs	0.28	_	DSF 23.9 ± 0.1 μg/mL: 57.78 ± 0.34, UC-NPs 77.7 ± 1.4 μg/mL: 54.75 ± 1.24, C-NPs 93.8 ± 0.4 μg/mL: 47.5 ± 0.31
						Caco-2	0.05	_	DSF 39.6 ± 0.3 μg/mL: 53.62 ± 0.53, UC-NPs 97.9 ± 0.5 μg/mL: 53.49 ± 0.59, C-NPs 148.3 ± 0.1 μg/mL: 40.28 ± 0.24
Wang et al.	China	Non-small cell lung cancer	24 h	Control	_	A549	0.45	_	DSF-LP-PLGA-MP 1, 3, 5, 7days: 9.32, 27.1, 28.2, 49.18
Yang et al.	China	Breast cancer	24 h	Control	_	MCF-7	0.29	_	DSF 0.2 μM/CuCl_2_ 10 μM: 27.56, DSF 0.25μM/CuCl_2_ 10 μM: 86.8
Kim et al.	Korea	HER2-positive breast cancer	24 h	DMSO	_	SKBR3	3.16	_	Cu 1 μM: 2.91, DSF 1 μM: 2.6, DSF/Cu: 30.21
						BT474	2.49	_	Cu 1 μM: 2.88, DSF 1 μM: 8, DSF/Cu: 40.76
Sharma et al.	India	Prostatic cancer	48 h	Control	STA (3mM)	PC3	8.34±2.2	26.31±5.5	DSF 1 μM: 15.04±3.14, DSF 2 μM: 19.71±4.2, DSF 3 μM: 32.06±6.16
						DU145	13.67±2.66	41.31±4.47	DSF 1 μM: 10.89±1.56, DSF 2 μM: 42.81±4.56, DSF 3 μM: 47.23±4.85
Zhao et al.	China	Pituitary adenomas	24 h	Control	TMZ (100μM)	Pituitary adenoma cells	0.29±0.09	0.81±0.23	DSF 25 μM: 0.31±0.10, DSF 25 μM + TMZ 100 μM: 1.64±0.16
Zhang et al.	China	Hepatocellular carcinoma	24 h	Control	_	Hep G2 cells	1.3	_	DSF-S-LNCs (PH = 7.4) : 9.4, DSF-S-LNCs (PH = 6.5) : 16.5
Duan et al.	China	Breast cancer	24 h	Control	_	4T1	1.07	_	DSF 1 μg: 34.77, DnMs (DSF 1 μg): 34.37, DCM (DSF 1 μg): 41.11
Rezk et al.	USA	Ovarian cancer	72 h	Control	_	A2780DK	4.15	_	DSF 5 μM: 36.4
Dastjerdi et al.	Iran	Pancreatic cancer	24 h	Control	_	PANC-1	27	_	DSF 5 μM: 51, DSF 10 μM: 84, DSF 13 μM: 92
Han et al.	China	Pancreatic cancer	72 h	Control	_	SW1990	1.5	_	DDTC–Cu(I) 1 μM: 6.4, DDTC–Cu(I) 3 μM: 17.7, DDTC–Cu(I) 5 μM: 24.8
Cen et al.	USA	Melanoma	48 h	Control	BSO (100M)	C81-46A	12.057±0.72	13.194±1.11	DSF 50 ng/ml: 25.35 ± 1.21, DSF 50 ng/ml + BSO 100 M: 54.78 ± 2.83

*Abbreviations*: *DOX* Doxorubicin, *CIS* Cisplatin, *PTX* Paclitacel, *STA* Staurosporine, *TMZ* Temozolomide, *BSO* Buthionine-sulfoximine, *DnMs* DSF-loaded noncrosslinked micelles, *DCM* DSF-loaded redoxsensitive shell crosslinked micelle, *DSF-LP-PLGA-MP* Disulfiram-loaded porous PLGA microparticle, *UC-NPs* Uncoated NPs, *C-NP* Coated NPs, *DDTC–Cu(I)* Diethyldithiocarbamate-Cu(I)

**Table 2:** Effects of disulfiram on tumor inhibition rates from animal studies

Information of reference			Information of animals				Intervention and tumor inhibition rate					Toxicity evaluation	
Reference	Country	Tumor	Strain and gender	Old (weeks)	Weight (g)	Animal tumor model	Intervention methods	Negative control	Positive control	Treatment group	Inhibit Rate	Parameter	Outcome
Peng et al.	China	Lung cancer	Female Balb/C nude mice	4−5	18−22	1.0 × 10^6^ A549 cells, SC, right flank	Every 4 days with 4 times, iv	Saline	_	DSF 10 mg/kg + copper 1.5 mg/kg ig PNpL-DSF/Cu(II)/DDC (1:1, 1mg/kg)	TSR% = 16.6% TSR% = 51.6%	No significant weight loss	Low
Parikshit et al.	China	Breast cancer	Female Balb/C nude mice	4−5	18 ± 2	1.0 × 10^5^ 4T1 cells, SC, left armpit	Every 3 days with 6 times, iv	Saline	_	DSF 15 mg/kg DSF-NLC 15 mg/kg TPGS-DSF-NLC 15 mg/kg	TGI% = 8.49%. TGI% = 29.2% TGI% = 48.24%	No noticeable body weight loss	Safety
Ji et al.	China	Breast cancer	Female Balb/C nude mice	_	20 ± 2	8.0 × 10^5^ 4T1 cells, SC, right flank	Everyday with 2 weeks, iv or every day with 2 weeks, ig	Saline	PTX (8mg/kg) TSR% = 55.01%	DSF 20 mg/kg ig DSF-NSps 20 mg/kg ig DSF-NSps 20 mg/kg iv DSF-NSps 10 mg/kg iv DSF-NSps 5 mg/kg, iv	TSR% = 0% TSR% = 59.03% TSR% = 80% TSR% = 75.86% TSR% = 69.21%	Weight increased slightly	_
Zhou et al.	China	Liver cancer	KunMing mice	5–6	_	1.5 × 10^7^ H-22 cells, SC, left axilla	Every 3 days with 4 times, iv	Saline	5-FU (20 mg/kg ) TIR% = 47.4%	DSF NPs 3 mg/mL DSF NPs 40 mg/kg + Cu(OI)2-S 0.3 mg/kg DSF NPs 40 mg/kg + Cu(OI)2-L 0.3 mg/kg	TIR% = 26.8% TIR% = 35.5% TIR% = 50.3%	_	_
Tao et al.	China	Breast cancer	Female Balb/C nude mice	_	20 ± 2	3.0 × 10^6^ 4T1 cells, SC, right flank	Every 2 days with 4 times, iv	Saline	DOX (5 mg/kg ) TIR% = 68.27%	DSF 5 mg/kg DOX 5 mg/kg +DSF 5 mg/kg Co-NPs (DOX 5 mg/kg + DSF 5 mg/kg)	TIR% = 34.81% TIR% = 80.92% TIR% = 89.27%	No significant difference in body weight change	Safety
Song et al.	China	Lung cancer	Female Balb/C nude mice	6	20.0	2.0 × 10^6^ A549DDP cells, SC, right flank	Every 2 days with 4 times, iv	Saline	_	PGA-CisPt 5.0 mg/kg PGA-CisPt 5.0 mg/kg+ NPs-DSF 10.mg/kg	TSR% = 45.6% TSR% = 75.4%	No body weight changes	Safety
Hamidreza et al.	Iran	Breast cancer	Female Balb/C nude mice	5	_	1.0 × 10^6^ 4T1 cells, mammary fat pad	2 weeks, iv	Blank NPs	_	DFS 10 mg/kg DS-P-NPs 10 mg/kg DS-PPF-NPs 10 mg/kg	TSR% = 17.07% TSR% = 66.67% TSR% = 75%	DS-P-NPs, DS-PPF-NPs groups more reduction weight than the DSF	No sign
Song et al.	China	Breast cancer	Balb/C mice	5-6	_	2.0 × 10^6^ 4T1 cells, SC, right flank	Every 2 days with 6 times, iv	Saline	_	DSF 15 mg/kg NP4/5/1 15 mg/kg	TSR% = 0 TSR% = 43.2%	No obvious body weight loss	Safety
Jennifer et al.	USA	Breast tumor	Female SCID mice	_	_	1.0 × 10^6^ SUM149 cells, SC, flank	Daily, iv	Vehicle	_	DSF 50 mg/kg DSF 50 mg/kg + Cu 0.5 mg/kg	TIR% = 75% TIR% = 84%	No noticeable body weight change	_
Choi et al.	Korea	Atypical teratoid/rhabdoid tumors	Female Balb/C nude mice	7	_	1.0 ×10^4^ AT/RT cells, SC, _	Every 5 consecutive days with 3 weeks, ip	DMSO	_	DSF 100 mg/kg	TSR% = 72.25%	_	No major
Vino et al.	China	Malignant Pleural Mesothelioma	Female Balb/C nude mice	5	_	0.5 × 10^6^ AB12 cells, SC, right flanks	Daily with 17 days, ip	Vehicle	_	DSF/Cu 50 mg/kg	TSR% = 71.5%	Weight of DSF-Cu group was 75% lower than that of vehicle group	_

*Abbreviations*: *DOX* Doxorubicin, *Cis* Cisplatin, *5-Fu* 5-fluorouracil, *V* Volume, *L* Length=longest diameter of the tumor, *W* Width=shortest diameter of the tumor, *SC* Subcutaneous, *iv* Intravenous injection, *TGI* Tumor growth inhibition rate—TGI% = [(Vc1-Vt1)/(Vc0-Vt0)]×100%, *TIR* Tumor inhibition rate—TIR% = [(Vc-Vx)/Vc] ×100%, *TSR* Tumor suppression rate—TSR% = [(Vc-Vx)/Vc] ×100%, *Vc* Mean tumor volume of the negative control group, *Vt* Mean tumor volume of certain administration group, *Vc1* Mean tumor volume in the negative control group at the time of tumor extraction, *Vt1* Mean tumor volume in the treatment groups at the time of tumor extraction, *Vc0* Mean tumor volumes in the negative control group, *Vt 0* Mean tumor volumes in the treatment group, *NPs* Nanoparticles, *NSps* Nanosuspensions, *NLC* Nanostructured lipid carriers, *TPGS* D-alpha-Tocopheryl polyethylene glycol succinate, *PNpL-DSF/Cu* Polymeric nanoparticles loading copper(II) diethyldithiocarbamate (DSF/Cu 1:1), *Cu(OI)2-S* Administration of copper oleate solution, *Cu(OI)2-L* Administration of copper oleate liposome, *NP4/5/1* The feed ratio of mPEG-PLGA/PCL/DSF was 4/5/1 in mass, *PLGA* Poly(lactide-co-glycolide), *PEG* Poly(ethyleneglycol), *mPEG-PLGA* Methoxy poly(ethylene glycol)-b-poly(lactide-co-glycolide), *PCL* Polycaprolactone, *DCC* N,N′ -Dicyclohexylcarbodiimide, *DCM* Dichloromethane, *NHS* Sulfo-N-hydroxysuccinimide, *DS-PPF-NPs* Disulfiram encapsulated PLGA PEG-folate NPs, *DS-P-NPs* Disulfiram encapsulated PLGA NPs

**Table 3:** Effects of disulfiram on progression-free survival and overall survival from human studies

Reference	Country	Study design	Study participants	Study protocol	OS	PFS	Adverse events
Huang, et al.	USA	Phase II, open-label, single-arm study	Recurrent GBM who had developed unequivocal progression after RT and concurrent TMZ as per the RANO criteria while receiving adjuvant TMZ or within 3 months from the last dose of TMZ”	DSF 80 mg and Cu Gluconate1.5 mg TID by mouth approximately 4–8h apart.	7.1 months (95% CI 5.8–8.5)	1.7 months (95% CI 1.4–1.9)	Nausea/vomiting (17%) followed by dizziness (9% grade). Only one patient (4%) had a possible DLT with grade 3 elevated alanine transaminase on day 31, which required study therapy to be held. The liver function test subsequently recovered after 4 weeks.
Huang, et al.	USA	Phase I, open-label, single-arm, single-institution study	Adjuvant TMZ in newly diagnosed adult GBM patients after standard chemoradiotherapy	7 patients at DSF 500 mg per day 5 patients at DSF 1000 mg per day, 6 patients at DSF 500 mg per day with Cu 2 mg	14.0 months (95% CI 8.3–19.6)	4.5 months (95% CI 0.8–8.2)	One with delirium after 1.6 months (without Cu), one with motor neuropathy after 2.6 months (without Cu) and one with diarrhea and nausea after 0.5 months (with Cu). All symptoms resolved shortly after dose reduction.
Nechushtan, et al.	Israel	Phase II, multicenter randomized double-blinded study	Newly diagnosed NSCLC patients were recruited. Patients with either stage IV or what was considered at the time “wet IIIb” (since 2009, these patients have been considered stage IV) were recruited. The patients were treated with only chemotherapy, and none were treated with either surgery or chemoradiation.	controls: six cycles of cisplatin and vinorelbine (plus placebo tablets), experimental groups: the same plus disulfiram (40mg three times daily).	10.0 versus 7.1 months	5.9 versus 4.9 months	_

*Abbreviations*: *GBM* Glioblastoma, *NSCLC* Non-small cell lung cancer, *TMZ* Temozolomide, *TID* Three times per day, *DLT* Dose-limiting toxicity, *RANO* Radiologic Assessment in Neuro-Oncology

Of 11 animal studies, Balb/C nude mice were utilized in nine [28–36], whereas the remaining studies used KunMing or female SCID mice [37, 38]. Ten studies used subcutaneous tumor models by injecting cancer cell lines [26, 29, 31, 32, 34–38], and one study used an in situ tumor model [33]. Eleven studies had assessed the dimensions of tumor volume (*V*) using the same formula (*V* = 0.5 × length × width^2^) [28–38], nine studies assessed changes in body weight in mice [26–34, 37, 38], and six studies contained data regarding the toxicity of DSF [28, 29, 32–35]. In addition, eight of the animal studies used DSF by re-synthesizing the molecule with nanomaterials [28–34, 37].

The three human studies included participants with differing characteristics and cancer types. All three clinical trials investigated DSF as a combination therapy with chemotherapy or/and radiation therapy [39–41], while two studies reported on adverse events [39, 40].

### Outcomes

Three cell lines and one animal study showed that treatment with DSF as a single agent induced apoptosis and increased the rate of tumor inhibition [17–19, 35]. Although the sensitivity between the various cell lines varied, dose-dependency was consistently observed.

The concentration-dependent increase in apoptosis and tumor inhibition rates was augmented by a combination therapy of DSF adding metal ions [copper (Cu), silver (Ag)] in 10 in vitro [8–11, 13–15, 20, 26, 42] and three in vivo studies [36–38]. The synergistic effect of Cis, DOX, TMZ, PTX, Gy, and DSF in induced apoptosis was significantly higher than that of DSF or Cis or DOX or TMZ or Gy alone [8–10, 21, 24, 42]. Tumor cell growth was significantly inhibited when DSF, chemotherapy, and radiation therapy were used simultaneously, as shown in the examined in vivo studies [30, 31, 35, 37].

Compared with free molecule, DSF encapsulated with nanomaterials significantly induced selective death-dependent apoptosis, especially in acidic conditions (pH = 6.5) in cancer cell lines. Eleven animal studies demonstrated that DSF modified by particular nanomaterials increased the tumor inhibition rate and that the anticancer activity was more obvious when chemotherapy (Cis) was combined with nanoencapsulated DSF [32].

Changes in body weight during the whole study period were analyzed in nine animal studies. With the exception of three reports of weight changes in DSF-treated or DSF-modified groups [30, 33, 36], other studies recorded that there was no noticeable body weight loss after DSF treatment or no significant difference in body weight changes across different groups [28–32, 34, 36, 38], which indicated that there was no major toxicity of DSF [28, 29, 32–35].

Many clinical trials have mentioned the use of DSF for solid tumors (www.clinicaltrials.gov). One study clearly analyzed the difference in PFS (5.9 versus 4.9 months) and OS (10.0 versus 7.1 months) between control and experimental groups [42]. PFS and OS both improved in the experimental groups. Two studies described PFS and OS of the entire research cohort, and the treatment efficacy seemed to be in contrast to historical data [39, 40]. Our systemic review included two single-arm trials in glioblastoma (GBM) patients and a randomized controlled trial in NSCLC patients. Although the two single-arm clinical trials did not compare treatment with a control group, positive effects were observed; e.g., a 40-year-old woman with unmethylated isocitrate dehydrogenase wild-type GBM had good health without any signs of tumor recurrence 33 months after study initiation.

Among the reported adverse effects, none were serious, and they were of grades 2–3. Adverse effects were reported in two studies and included diarrhea, nausea, dizziness, vomiting, motor neuropathy, and elevated alanine transaminase levels. Symptoms resolved quickly when the dose was reduced [39, 40].

All three studies show that DSF is safe and seems to prolong survival of cancer patients. Because of individual differences in patients, the response to DSF was also varied [39, 40, 42]. The optimal concentration and sensitivity type should be further explored by in vitro and animal studies.

## Discussion

DSF is decomposed into diethyldithiocarbamate in the body and exhibits anticancer activities [43]. Considering that the loss of cellular proliferation control leads to the development of cancer, effective clinical therapies of cancer have been developed based on the principle of inducing apoptosis [44]. In the included animal studies, the tumor inhibition rate was utilized to evaluate antitumor efficiency by calculating tumor volume. Most studies included in this review revealed enhanced apoptosis and tumor inhibition rates with DSF treatment (Table 4).

**Table 4:** The summary of the findings

	Studies	Evaluation indicator	Results	Side effects
Cells studies	21	Apoptosis rate	From 4.8 to 92%	N/A
Animals studies	11	Tumor inhibition rate	From 8.49 to 89.27%	Safety
Human studies	3	PFS and OS	Be prolonged	Low

In recent years, metal-based complexes have been reported to exhibit anticancer activity [45]. Silver complexes demonstrate anti-tumor activity and display low toxicity in humans. The mechanism of action is related to their interaction with nucleic acids and proteins [46]. Metabolites of DSF chelate with metal ions, leading to alterations in the intracellular levels of metal ions, enhancement of oxidative stress, inhibition of the activities of superoxide dismutase or matrix metalloproteinases, inactivation of essential sulfhydryl groups by protein carbamoylation, and alteration of cancer cell invasion, tumor angiogenesis, and metastasis [47, 48]. The observation that the combination of DSF with metal ions (Cu, Ag) leads to enhanced anticancer effectiveness is in accordance with the observations of in vitro and animal experiments [11, 14].

In different cancer cell lines, the lethal concentration of DSF was different. The lethal concentration was reduced when DSF combined with metal ions or nano-reconstructed DSF.

The additive/synergistic action of DSF with other chemotherapy agents in inhibiting tumor cell growth and cytotoxicity is mediated through the enhancement of cellular oxidative stress, inhibition of P glycol-protein (P-gp) activity, and dysregulation of the NF-κB signaling pathway [8, 49, 50].

In the examined studies, anti-tumor activity, as evidenced by higher apoptosis and tumor inhibition rates, was enhanced with DSF-NPs in various ways. At the pH of 7.4, the half-life of DSF is 1–1.5 min [47]. The half-life was improved by nanomaterial packaging of DSF, with the anti-tumor effects increasing under acidic conditions (pH = 6.5) [51]. DSF-NPs enhanced cellular uptake, induced high levels of reactive oxygen species, activated the MAP-kinase pathway, sustained drug supply, and blocked copolymer micelles, such as the P-gp inhibitor [14, 20, 52]. Evidence supports that DSF-NPs ameliorate the instability and low treatment efficacy of free DSF.

Event-free survival (EFS) means that there are no adverse events since the start of treatment, including change of regimen, adverse side effects, intolerance, disease progression, and patient death. EFS represents a direct measure of the ability of the treatment to achieve a response, the durability of the response achieved, and its capacity to prolong life [53]. It was found that the doses of disulfiram significantly increased EFS [39].

Although our results may be more reliable than those of single studies, the present study has certain limitations. First, only articles published in English and Chinese were included; the non-inclusion of articles published in other languages may have had an effect on the results. Second, only some solid tumors were included, not referred to non-solid tumor (hematological malignancy). Third, the scarcity of the studies in general (35 in total) and the fact that they are performed on different cancers may make any specific conclusions difficult. Finally, no quality evaluation was conducted, and the majority of studies were animal and cell experiments; thus, the translation of these results to benefits in the clinic needs to be determined.

In conclusion, many studies have investigated the antineoplastic activity of DSF. This systematic review provides evidence of the antineoplastic activity of DSF in vitro, in in vivo animal models, and in humans. DSF could induce cancer cell apoptosis in cell experiments and inhibit cancer cell growth in animal experiments. Administration of DSF as a combination therapy or as a nanoparticle-encapsulated molecule seems to enhance its effectiveness. Meanwhile, DSF hardly affect the animal weight. Above of all, DSF is effectiveness and safety. These findings may serve as the basis for designing clinical studies of DSF in the future.

## Supplementary Information

**Additional file 1.**

## Data Availability

Not applicable.

## Abbreviations

DSF: Disulfiram
PFS: Progression-free survival
OS: Overall survival
EFS: Event-free survival
TIR: Tumor inhibition rate
