# Supplementary figures and images for: Anticancer effects of disulfiram: a systematic review of in vitro, animal, and human studies
Source: Syst Rev. 2022 Jun 2;11:109. doi: 10.1186/s13643-021-01858-4 (PMC9161604; doi:10.1186/s13643-021-01858-4)

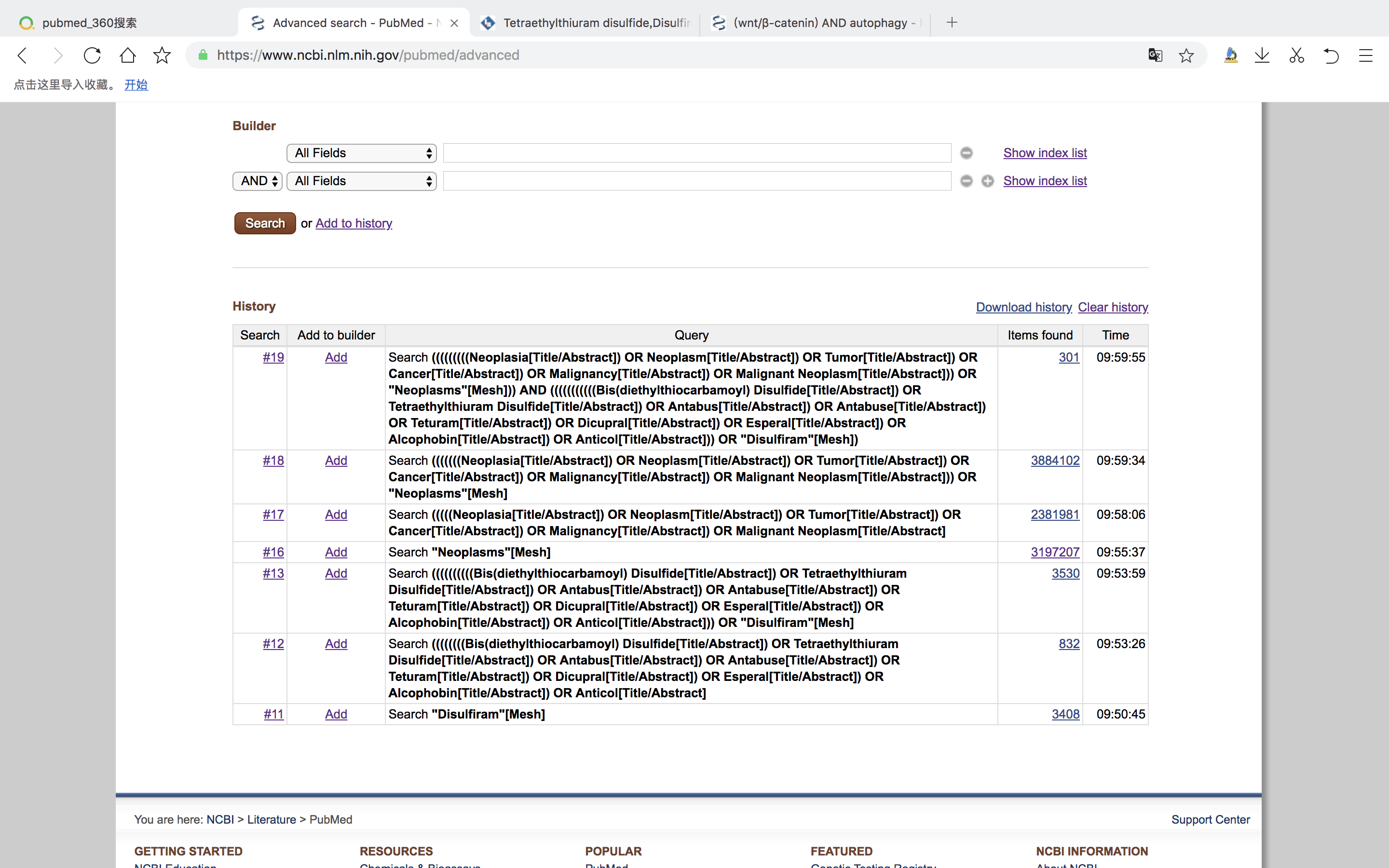

Supplement: Supplementary file 1 — Additional file 1. [file 13643_2021_1858_MOESM1_ESM.zip › 1.png]

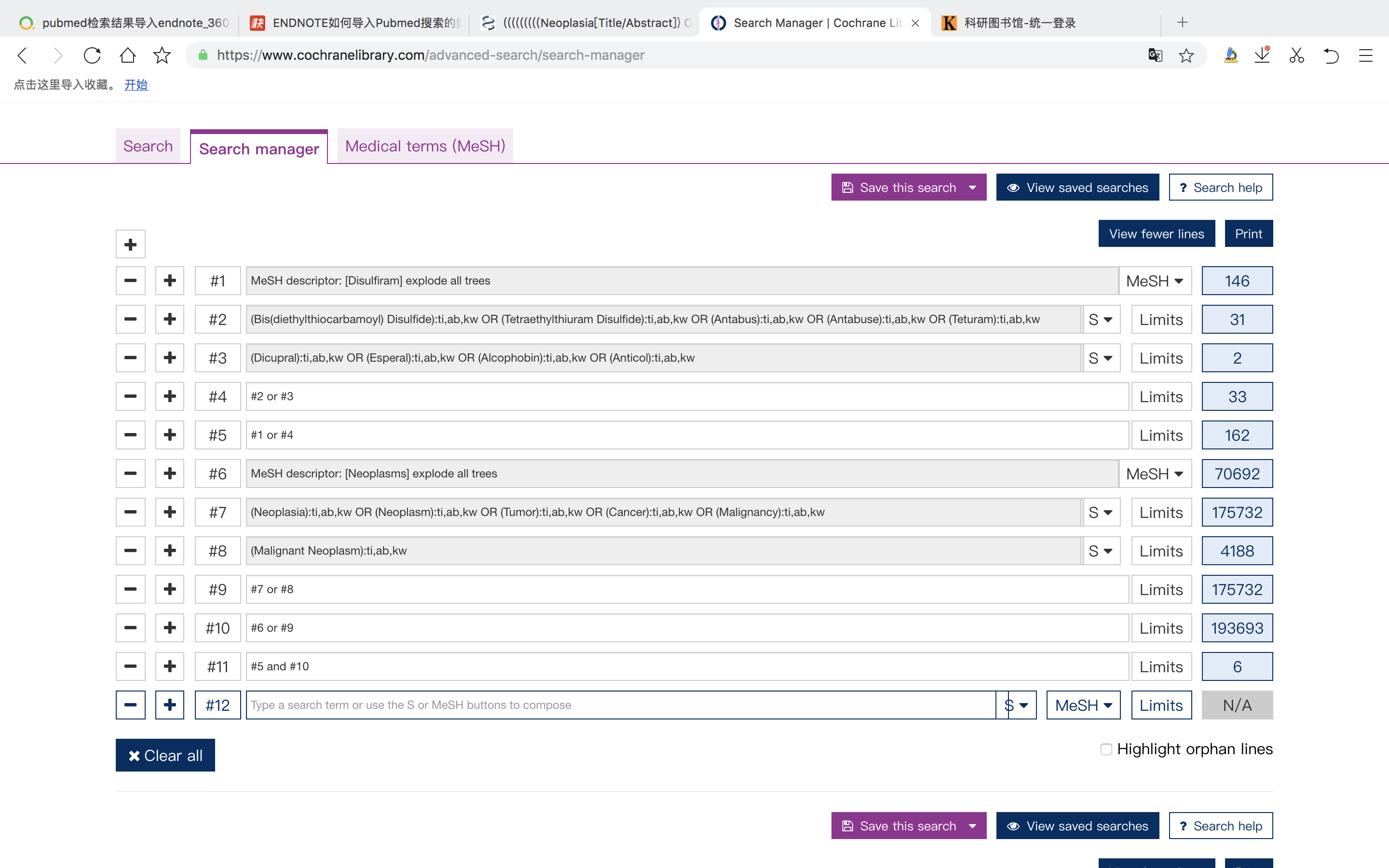

Supplement: Supplementary file 1 — Additional file 1. [file 13643_2021_1858_MOESM1_ESM.zip › 2.png]

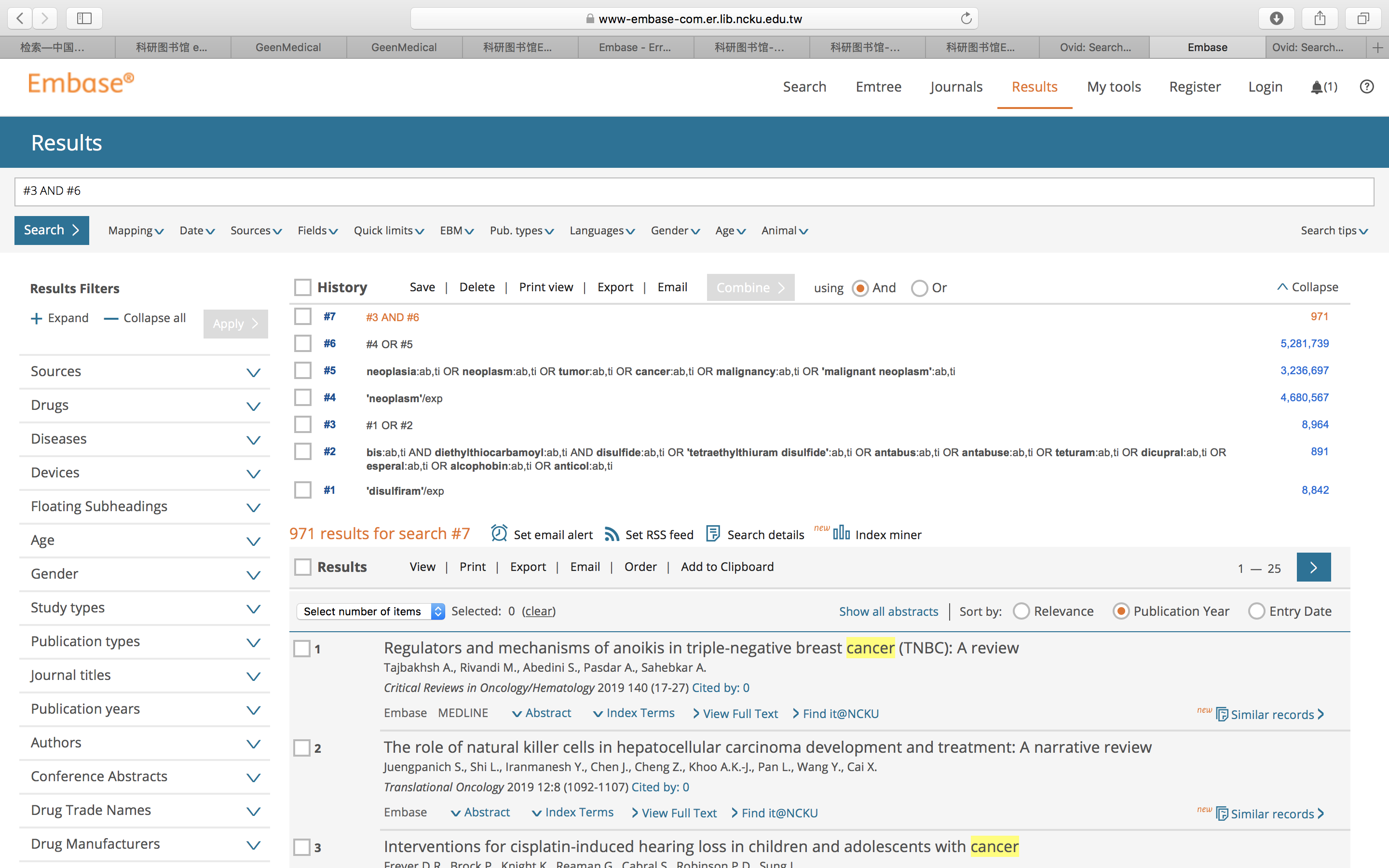

Supplement: Supplementary file 1 — Additional file 1. [file 13643_2021_1858_MOESM1_ESM.zip › 3.png]
